# Distinguishing critical microbial community shifts from normal temporal variability in human and environmental ecosystems

**DOI:** 10.1038/s41598-025-01781-x

**Published:** 2025-05-15

**Authors:** Ann-Kathrin Dörr, Sultan Imangaliyev, Utku Karadeniz, Tina Schmidt, Folker Meyer, Ivana Kraiselburd

**Affiliations:** 1 Department of Medicine, Institute for Artificial Intelligence in Medicine, University Hospital Essen, University of Duisburg-Essen, Essen, Germany; 2 Department of Computer Science, University of Duisburg-Essen, Essen, Germany; 3 Emschergenossenschaft/Lippeverband, Kronprinzenstraße 24, 45128 Essen, Germany

**Keywords:** Time series prediction, OneHealth, Machine learning, Outlier detection, Early warning, Computational models, Microbial communities

## Abstract

Differentiating significant microbial community changes from normal fluctuations is vital for understanding microbial dynamics in human and environmental ecosystems. This knowledge could enable early warning systems to monitor critical changes affecting human or environmental health. We applied 16S rRNA gene sequencing and time-series analysis to model bacterial abundance trajectories in human gut and wastewater microbiomes. We evaluated various model architectures using datasets from two human studies and five wastewater settings. Long short-term memory (LSTM) models consistently outperformed other models in predicting bacterial abundances and detecting outliers, as measured by multiple metrics. Prediction intervals for each genus allowed us to identify significant changes and signaling shifts in community states. This study proposes a machine learning model capable of monitoring microbial communities and providing insights into their responses to internal and external factors in medical and environmental settings.

## Introduction

The gut microbiome has a significant influence on the development of various diseases. In some cases, such as inflammatory bowel disease^1,2^ or obesity^3,4^, the connection is well-established. Emerging evidence also suggests links, even though more subtle, between microbiome dynamics and neurodegenerative diseases^5–11^. Research continues to explore these links, potentially paving the way for new treatment approaches.

Defining a core or “healthy” set of microbes, and thus establishing a healthy microbiome state, is a key focus in microbiome research and has been explored by various research groups. Yet the notion of a stable, “healthy” microbiome remains elusive, given that microbial communities fluctuate over time in response to diet, lifestyle, and host physiology^12–18^.

**Fig. 1:**
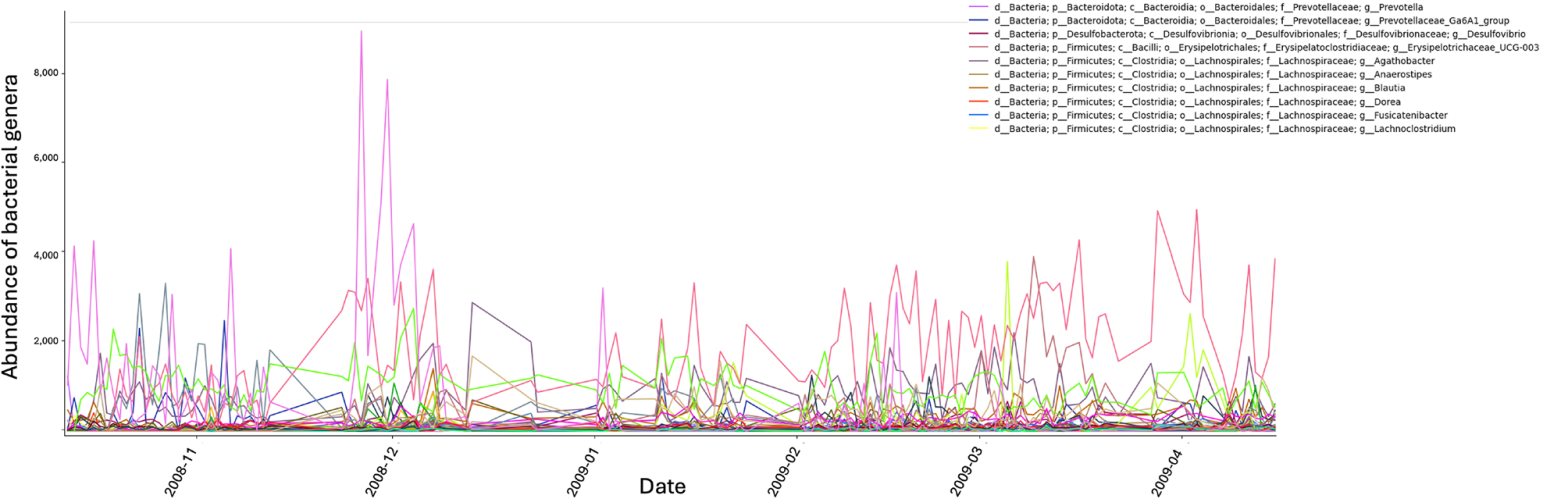
Abundance of bacterial genera over time found in the human gut microbiome. Data analyzed correspond to one participant from Caporaso *et al.*^16^. Fluctuations in absolute abundances occur at different time points and for all genera. The different genera are depicted in the graph in different colors. For simplification, the legend shows the ten most abundant genera.

Early frameworks, such as enterotypes^12,13^, sought to classify gut microbiota into discrete community configurations. While these clusters give valuable information in a medical context for evaluating the connection between the microbiome and diseases, they are undercomplex when it comes to time-dependent settings. Microbial communities in human and environmental contexts are dynamic^19^, with temporal fluctuations and data variability being common in microbial community analyses, as illustrated in Fig. 1. The figure presents a typical example of microbial abundance variation over time in the human gut microbiome. While simple statistical methods may suffice for analyzing a single bacterial taxon, evaluating all taxa across numerous time points quickly becomes challenging. Visual inspection and ad-hoc statistical approaches, without accounting for normal fluctuations, often fail to reliably detect outliers or significant changes. Advanced computational approaches, including machine learning (ML) and time series models, can integrate multi-dimensional data, leverage temporal correlations, and accommodate non-linear relationships^20,21^, like those expected to be found in microbial data. For these reasons we propose to employ machine learning to effectively analyze and predict microbial community changes.

Changes in microbiomes over time and across locations can be effectively monitored using DNA sequencing^22^. However, sequencing processes are prone to system-specific errors^23^, along with variability introduced by extraction protocols and sample handling^24^. Addressing these errors requires computational analysis pipelines such as RiboSnake, Natrix, or Tourmaline^25–27^. Microbiome data is typically represented as a sparse matrix containing information on the abundance of various entities, with the BIOM^28^ standard providing a suitable format for data storage and exchange. These datasets often include dozens to hundreds of time points and hundreds of thousands of entities with their respective abundances. Extensive research has been conducted on preprocessing this data for downstream analysis^25,29–31^, including debates about normalizing the inherently noisy data and whether to represent microbial diversity using ASVs (amplicon sequence variant) or OTUs (operational taxonomic unit)^32^.

Several approaches have been proposed for modeling relationships between bacterial species within a system, often incorporating additional variables. Generalized Lotka-Volterra models have long been utilized for this purpose^20,33^. Another approach involves modeling entire microbial communities while accounting for correlations using mathematical descriptions of community variability^34^. For time-series forecasting, the autoregressive integrated moving average (ARIMA) model is a widely used technique^35^ and has proven effective across many applications^36–38^. However, simple ARIMA models are limited in handling seasonal or multivariate data^39^. To predict bacterial abundances over extended periods across multiple species, models like vector autoregressive moving average (VARMA)^40^, a variation of ARIMA, are preferred. Random Forest (RF) regressors, introduced in 2001^41,42^, are another well-established machine learning method, known for their effectiveness in time-series prediction and their ability to outperform ARIMA models in some cases^43,44^. RF models are also frequently used for analyzing feature importance, which could provide insights into the roles of different bacteria in abundance prediction^45^. Long Short-Term Memory (LSTM) networks have also demonstrated strong performance in microbial time-series analysis, as shown in studies by Baranwal *et al.*^21^ and Jang *et al.*^46^. LSTMs are particularly suited for tasks requiring retention of past information for future predictions due to their architecture allowing connections between hidden units over time delays^47^, an idea first proposed in the 1980s^48^. Originally designed for Natural Language Processing, LSTMs are now widely applied to various time-series tasks^49,50^. Gated Recurrent Unit (GRU) models, another type of Recurrent Neural Network with fewer parameters, are also an option for time-series prediction^51^.

In this study, we investigate time-series analysis and machine learning as an approach for microbiome-related predictions, incorporating both the microbiome’s temporal variability and overall stability. The aim is to develop a model that can serve as a basis for an early warning system, distinguishing typical fluctuations from significant microbial changes that may signal potential risks. This model could have practical applications, such as monitoring microbiome changes in hospitalized patients, particularly those in intensive care units (ICUs), who often experience significant alterations in their gut microbiome^52,53^. ICU patients are at a heightened risk of developing sepsis, a serious condition where the body’s response to infection leads to widespread inflammation, potentially resulting in tissue damage, organ failure, or death^54^. Identifying critical changes in microbiome trajectories could be especially valuable for conditions like sepsis, where early detection and intervention significantly impact patient outcomes^55,56^.

Beyond clinical settings, the model could also be applied to studying microbial communities in complex environments, such as wastewater, which can serve as proxy for community-level health status and can allow tracking emerging pathogens or stress conditions. Recent public health challenges, including the SARS-CoV-2 pandemic^57^, have emphasized the connections between human, animal, and environmental health^58^. This has further highlighted the role of wastewater epidemiology in forecasting outbreaks and identifying emerging pathogen variants^59^. As environmental sequencing data becomes increasingly available, the ability to distinguish normal biological variations, such as those driven by seasonal or temperature changes, from irregularities becomes essential. Monitoring shifts in microbial abundance in wastewater could support early warning systems for detecting potential pathogen growth in populations^60^, which is critical for addressing public health challenges^61^ and climate change impacts^62^.

With these applications in mind, we assessed the performance of various predictive models, using a Vector Autoregressive Moving-Average (VARMA) model as baseline. This approach was applied to 16S rRNA gene amplicon sequencing datasets from both human microbiome studies and wastewater samples to evaluate its utility in medical and public health contexts.

## Data

### Human microbiome data

The human microbiome data used for training and testing the machine learning models were sourced from two previous studies. The first dataset, from Caporaso *et al.*^16^, includes 16S rRNA gene amplicon sequencing data collected over 396 time points. Samples were taken from two healthy participants (one male and one female) using swabs for their palm and tongue microbiomes, as well as stool samples representing the intestinal microbiome. This dataset only included metadata on gender and sampling time, with no additional details. The study provides a large longitudinal time series with just a small amount of missing data points. With the samples taken from multiple body sides, it provides a comprehensive view of the microbiome.

The second dataset contains 16S rRNA gene amplicon sequencing data from two male participants of different ages, with stool and saliva samples collected over a year. Unlike the first dataset, this study provides information on abnormalities in the participants’ health status as well as some dietary information^63^.

These two studies were chosen as they provide the longest and most frequently sampled time series data available for human-associated microbiome.

### Environmental microbiome

To train the machine learning models on environmental data, we used samples from the inlet of four wastewater treatment plants (WWTPs) from two existing studies and data we generated during weekly monitoring of a local WWTP (Table 1).

The first study analyzed wastewater from the Chicago area^64^, and the second focused on WWTPs in Milwaukee^65^. Both datasets included several years of monthly sampling, but the Chicago dataset was incomplete, missing data for December through February across all years, along with additional gaps. For the Chicago samples, metadata such as precipitation and median temperature were retrieved from Visual Crossing weather data services^66^. The Milwaukee dataset provided more complete information, including precipitation, temperature, and other parameters. However, disparities were observed between the two sampling sites, such as differences in the number of missing samples and sampling duration. The Jones Island site in Milwaukee was selected as the primary dataset for training and evaluating the machine learning models due to its comprehensive metadata and sampling points. Both datasets were chosen for their long-term sampling, allowing the incorporation of seasonal patterns into the training data. With the supplementary metadata of the Milwaukee dataset, the influence of environmental factors on the models’ prediction could be further analyzed.

The third 16S rRNA gene amplicon sequencing dataset was generated from a year of weekly sampling at the inlet of a WWTP in Dinslaken (KLDI), located in the Ruhr area of Germany. This plant serves approximately 69,480 residents and two hospitals. The sampling strategy followed the method described by Schmiege *et al.*^59^, collecting 2 L of untreated wastewater. Samples were stored at 4$^{\circ }$C until further processing, when aliquots of 200 mL were filtered using electronegative filters with 0.45 µm pore size (MF-Millipore). Afterward, nucleic acid extraction was done using the innuPREP AniPath DNA/RNA Kit on an InnuPure C16 touch device (Analytik Jena). Sequencing libraries were prepared according to the 16S Metagenomic Sequencing Library Preparation protocol (Illumina), using the primers Bakt_341F and Bakt_805R targeting the V3-V4 region^67^. Libraries were sequenced on an Illumina MiSeq instrument using the 2x250 V2 chemistry.

Data has been deposited under the project accession PRJEB83882.

**Table 1:** Overview of wastewater data used in this study.

Name	Sample description	Reference	URL
MIL	Monthly data 2013–2018 from Milwaukee WWTP	^64^	https://www.ebi.ac.uk/ena/browser/view/PRJEB51632
CHI	Monthly data 2014–2019 from Chicago area WWTP	^65^	https://github.com/NewtonLabUWM/Sewage_TimeSeries/tree/master/RData
KLDI	Weekly data from WWTP Dinslaken, Germany	PRJEB83882	https://www.ebi.ac.uk/ena/browser/view/PRJEB83882

## Methods

Since the microbiome data used in this study originated from multiple sources, including previously published studies and self-generated data, we standardized all input data before training the machine learning models. For this, we re-analyzed all fastq files with RiboSnake^25^ a 16S rRNA gene amplicon sequences analysis pipeline based on QIIME2^29^.

The analysis includes quality and abundance filtering, clustering, classification, and rarefaction. For this work, we employed the parameters listed in the respective GitHub repository^68^. We performed an analysis based on OTUs, with taxonomic information given at the genus level for all samples. Analysis based on ASVs yielded no significant differences. Taxonomic classification was performed based on SILVA version 138^69^, although analysis with Greengenes 2^70^ has given similar results.

The generated feature table holding the absolute abundances (number of reads per OTU) of the bacterial genera in the different samples was normalized with a scaler to fit the interval from 0 to 1 so that all inputs are transformed equally before being used as input for the machine learning models. With this data at hand, our goal was to create models generating a prediction interval that captures 95% of the expected abundance values. For the final visualization, the values were transformed back into absolute abundances.

For the human dataset, data points associated with reported illness on the sampling day were excluded to ensure the model was trained on data from healthy individuals.

Of the analyzed data, 80% was allocated for training and validation, while 20% was reserved for testing.

To compile the list of bacterial genera for training the models, all genera identified in the samples of the dataset were combined. Additionally, bacterial data from other relevant studies on significant taxa were included. To ensure the representation of key components of the human microbiome, the list was supplemented with the 109 baseline species of the human gut microbiome identified by King et al.^71^, with their taxonomy sourced from the SILVA database version 138^69^. Genera associated with sepsis, as reported by Nabizadeh et al.^72^, were also added.

Various model architectures were developed and tested to determine the most suitable approach for predictions (Table 2). Random Forest, LSTM, and GRU models were implemented using Keras^73^ and TensorFlow^74^, while the VARMA model was built with the Python library Statsmodels^75^. Model performance was assessed using Mean Absolute Error (MAE), Root Mean Squared Error (RMSE), and Normalized Root Mean Squared Error (NRMSE). In addition to these metrics, predictions were visualized by plotting actual abundance values of the bacterial genera alongside model predictions for the training, validation, and test sets, as well as prediction intervals. These plots were generated for each genus separately.

RF, LSTM, and GRU were chosen as tested model architectures, as different studies showed that they work well in different settings with microbiome data^21,76,77^.

### VARMA

ARIMA models are commonly used for time series predictions, particularly in economic applications^36,37^. To handle multivariate time series, ARIMA can be extended with a vector component, resulting in the Vector Auto-Regressive Moving Average (VARMA) model^40^. Ensuring stationarity of the data is crucial for this type of model. The Dickey-Fuller test^78^ was applied to each data column to check for stationarity. Since the data was found to be non-stationary, first-order differencing was used to address this. The parameters for the auto-regressive (p) and moving average (q) components were determined through hyperparameter optimization using a grid search across the parameter space^79^. The resulting VARMA (0,1,3) model included a linear time trend as a trend parameter. While ARIMA models provide greater interpretability due to their linear structure, they are limited in capturing complex relationships^80^.

### Random forest

Random Forest prediction relies on combining multiple decision trees^41^. Each tree is trained on a randomized subset of the data, a process known as bagging, which introduces variability and reduces correlations between trees^81^. This approach enhances the model’s robustness against noise and overfitting. Random Forests can be applied to both classification and regression tasks^82^. For this analysis, a Random Forest algorithm with 100 estimators was used, employing Mean Absolute Error (MAE) as the criterion for feature splitting. The method’s ability to handle both linear and non-linear relationships, along with its resistance to overfitting, makes it suitable for working with small microbiome datasets. However, for larger datasets, the computational demands increase as the number of trees grows and the required storage scales with tree depth^42^.

### LSTM

Long Short-Term Memory (LSTM) models are a specialized type of Recurrent Neural Network (RNN) designed to address the vanishing gradient problem that can occur during training. In standard RNNs, repeated backpropagation can lead to diminishing gradient values over time, reducing the network’s ability to learn long-term dependencies^83^. LSTMs overcome this issue by incorporating a gating mechanism that regulates the addition and removal of information from previous time steps^84^. For this study, LSTMs with varying numbers of cells and layers were implemented. The rectified linear unit (ReLU) activation function was used^85^, and dropout was applied to mitigate overfitting^86^. Additionally, early stopping was employed to stop training once the loss function stopped declining, helping to prevent unnecessary iterations. The models were optimized using the ADAM optimizer^87^ with Mean Absolute Error (MAE) as the loss function. Guided by insights from Jang *et al.*^46^, particular focus was placed on LSTM architectures with 2048 cells and different numbers of hidden layers. While LSTMs are well-suited for modeling long-term dependencies and are robust to noise, overfitting remains a potential challenge that must be managed carefully during training.

### GRU

Gated Recurrent Units (GRUs) are a type of Recurrent Neural Network (RNN) architecture introduced by Cho et al. in 2014^88^. Like Long Short-Term Memory (LSTM) networks, GRUs are designed to address the vanishing gradient problem that can occur in RNNs. GRUs differ from LSTMs in their simpler design, using two gates-an update gate and a reset gate-compared to the three gates in LSTMs (forget, input, and output gate). This reduced number of gates means GRUs require fewer hyperparameters, making them less complex and potentially less prone to overfitting while still effectively handling sequential data^51^.

**Table 2:** Characteristics of different model architectures tested for microbial abundance time series prediction.

	LSTM	GRU	RF	VARMA
Architecture	Sequential processing	Sequential processing, simplified design	Ensemble learning approach	Extension of ARIMA for multivariate time series
Effectiveness modeling long-term dependencies	High	Moderate to high	Moderate	Moderate to low
Advantages	Retention of past information	Retention of past information	Robust against overfitting	Complex relationships between variables
Disadvantages	Large datasets best	Large datasets best	Long computing times	Requires stationarity and linear structure

### Evaluation and prediction interval

To evaluate the performance of the different model architectures, training was conducted using only the female dataset from Caporaso et al.^16^. This approach allowed for the identification of the most suitable model for the task, based on the predefined evaluation metrics, while also benefiting from shorter training times. The female gut dataset was selected because it showed the fewest anomalies during visual inspection. The architecture that achieved the best results based on these metrics was then utilized for further training and analysis. Predictions were made iteratively, with each prediction step based on the three preceding steps.

For each genus, a prediction interval was established. This enabled the identification of outliers and the detection of potential health risks or environmental stressors. It was created by training multiple models of the same architecture on the same dataset. By assuming a normal distribution of predictions, we calculated 95% confidence intervals using the standard deviation and critical value z. This approach allowed us to compare observed abundances against the prediction interval’s upper and lower boundaries, enabling outlier detection (Fig. 8 in the Appendix)^89^. Outlier detection was performed by comparing observed bacterial abundances against the prediction interval. If a measured value fell outside the interval’s upper or lower boundaries, it was identified as an outlier.

### Feature interpretation

To understand model predictions, we applied SHAP analysis^90^ to quantify the influence of individual genera on predictions. Additionally, SCNIC correlation networks were generated to explore community structure^91^. This integrative approach allowed us to examine whether highly influential genera were also key networked taxa, providing ecological context to model-driven insights.

#### SHAP

The LSTM model results were further examined using Shapley Feature Importance (SHAP)^90^ to assess the significance of the input features. SHAP applies Shapley values and a game-theoretic approach to quantify the contribution of individual features to the model’s predictions and has proven effective for analyzing time-series data^92^. The bacterial genera were ranked based on their positive and negative influence on the model’s performance, as indicated by the computed Shapley values. The findings were visualized using SHAP’s built-in plotting tools^93^.

#### SCNIC

The results of the SHAP feature importance analysis were compared with those generated by the network analysis tool SCNIC^91^. SCNIC, which operates on 16S rRNA gene data, was utilized through its QIIME2^29^ implementation. It computes network correlation metrics for all bacterial genera in the dataset or predefined bacterial clusters. These correlation networks provide insights into the relationships among different bacterial genera^91^. Correlation interaction networks were generated using SCNIC’s four available metrics: Spearman’s $\rho$, Pearson’s r, Kendall’s $\tau$, and SparCC^94^, applied to all samples from all individuals.

## Results

In this work, we employ time series datasets containing abundances of bacterial genera based on 16S rRNA gene amplicon sequencing. These were used for training, validating, and testing various machine learning techniques for predicting bacterial abundances and detecting outliers. The datasets included human and wastewater samples, as detailed in the methods section. Each selected machine learning method demonstrated suitability for the tasks, though with varying levels of accuracy. Initially, a subset of the data was used to identify the most effective model architecture for accurate predictions. Once the optimal model was identified, it was trained on the complete dataset. This approach minimized the time required for training, validation, and testing during the initial evaluation phase.

### Results for microbial communities from human origin

For human-associated data, initial training and evaluation were performed using intestinal microbiome data from the female participant described in the data section. After identifying the most effective architecture, the model was trained on the complete dataset, which included intestinal microbiome data from four individuals. A data frame with 225 bacterial genera was used as input, where genera absent from an individual’s samples but present in others were assigned a value of zero.

The evaluation metrics for the models are summarized in Table 3. Among the tested models, the LSTM showed the best performance, while the baseline VARMA model demonstrated the poorest prediction accuracy, with the worst values for the evaluation metrics. The Random Forest and most GRU models tended to overfit. This is indicated by evaluation metrics for the test set being more than three times higher than those for the training set^95^. When examining different LSTM configurations, models with 8 to 32 cells did not overfit but had limited predictive capabilities. In contrast, models with more cells exhibited some overfitting but showed improved accuracy in predicting short-term variations. Given these results, the LSTM architecture that initially showed signs of overfitting but provided better prediction accuracy was selected for further training.

We hypothesized that increasing the amount of training data could reduce or eliminate overfitting in the selected model. This hypothesis was confirmed as increasing the dataset size (by integrating additional subjects) reduced overfitting and improved generalization. Table 4 shows that overfitting was nearly eliminated.

To validate that the evaluation metrics consistently identified the best-performing model across all human datasets, the model was trained separately on each subset. The results confirmed that the LSTM consistently delivered the best overall performance, as detailed in Table 7 in the Appendix.

**Table 3:** Table depicting the results for the different evaluation metrics. Models have been trained on a smaller training dataset containing the intestinal microbiome data of the female subject from Caporaso et al.^16^. All LSTMs were created with dropout and used early stopping. A tendency to overfit can be detected, as the evaluation metrics for many models are more than two times higher for the test than for the training data. However, as the training was done on a subset only, the overfitting might be tackled when training on all data points. With this in mind, the comparison of the different evaluation metrics emphasizes that the LSTM with one layer and 2048 cells works best.

Architecture	MAE training	MAE test	RMSE training	RMSE test	NRMSE training	NRMSE test
LSTM (1 layer, 8 neurons)	194.36	248.23	755.35	873.88	0.24	0.27
LSTM (1 layer, 16 neurons)	170.89	263.24	672.12	952.93	0.21	0.29
LSTM (1 layer, 32 neurons)	134.27	277.08	531.07	976.25	0.16	0.29
LSTM (1 layer, 2048 neurons)	21.33	217.56	72.50	789.20	0.02	0.25
LSTM (2 layers, 2048 neurons)	37.56	222.47	126.06	842.85	0.04	0.25
LSTM (3 layers, 2048 neurons)	38.48	268.25	149.64	1013.43	0.05	0.28
GRU (1 layer, 4 units)	198.9	286.86	721.27	886.97	0.23	0.27
GRU (1 layer, 16 units)	128.81	505.26	391.25	2088.95	0.12	0.5
GRU (1 layer, 1024 units)	22.53	343.25	64.10	1212.34	0.02	0.35
Encoder–decoder	159.53	254.09	621.27	869.41	0.19	0.28
Random forest	89.43	217.26	299.20	772.64	0.09	0.24
VARMA	540.61	592.57	1844.06	1710.53	11.39	0.38

**Table 4:** Values of all validation metrics for the different datasets employed for training the LSTM model. A one-layer model with 2048 LSTM cells was employed. The model’s predictions tend to decline in accuracy with additional data but at the same time, the overfitting of the model is greatly reduced. The higher values for the evaluation metrics could also be a result of higher bacterial abundances in the third and fourth dataset.

Metric	Female	Male	DonorA	DonorB
MAE train	3.89	6.91	173.85	94.20
MAE test	45.61	20.79	520.78	257.18
RMSE train	40.25	65.51	1542.17	1044.42
RMSE test	381.61	106.93	3467.74	2347.96
NRMSE train	0.03	0.55	0.28	0.18
NRMSE test	0.32	1.30	1.01	0.48

Training multiple LSTM models allowed estimation of 95% prediction intervals, successfully encompassing the majority of observed abundances. Deviations beyond these intervals flagged potential outlier events.

**Fig. 2:**
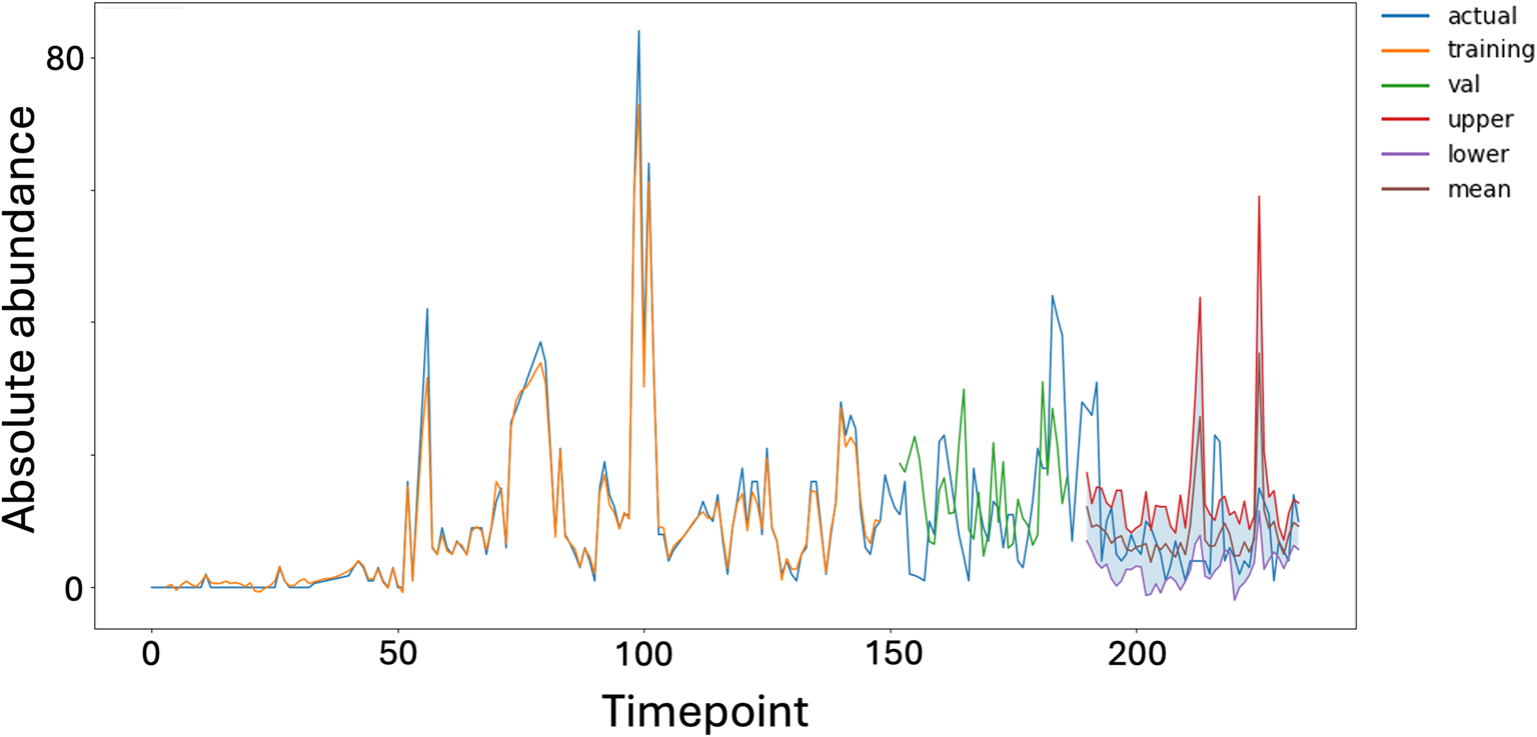
Abundance over time of genus *Coprobacter* in the intestinal microbiome dataset corresponding to the male subject from Caporaso et al.^16^. Result after retraining the model, previously trained on the dataset corresponding to the female subject. Orange shows the predictions on the training set, green the predictions on the validation set, and brown the mean prediction for the test data calculated from all predictions of the 50 models used for prediction interval creation. Only a few values of the test dataset are not covered by the 95% prediction interval. This is essential for outlier detection in the future.

To establish the prediction interval, 50 independent LSTM models with the same architecture were trained on the dataset. A standard normal distribution was assumed for the prediction outputs. A 95% prediction interval was calculated using the standard deviation and the critical value z, defining the upper and lower boundaries. The final prediction was obtained as the average of all model outputs. Outliers were identified by comparing the actual measured abundances with the interval boundaries. Figure 2 provides an example of the prediction results and interval for a specific bacterial genus, demonstrating that nearly all measured abundance values fall within the interval.

The prediction interval is critical for outlier detection, but understanding the importance of input parameters can provide insights into the influence of specific genera and their changes under the studied conditions. In deep learning models, interpretability often poses a challenge. Assessing whether the bacterial genera identified as most influential in the model’s predictions align with key genera in the broader bacterial community composition could help clarify some of the model’s outcomes.

When analyzing the model’s predictions for individual bacterial genera, we observed that the prediction accuracy varied across genera, with some being predicted less accurately than others (Appendix Fig. 7). To identify potential issues in the data that might reduce prediction accuracy, we calculated MAE, RMSE, and NRMSE for each genus across all subjects. MAE and RMSE are calculated in the same range as the abundance values, while the NRMSE normalizes the RMSE by dividing it by the standard deviation of the predicted values. This normalization makes the NRMSE more appropriate for comparing prediction errors across models and datasets, as it is independent of the scale of the prediction values. For instance, predictions for genera with high abundance can be compared to those with low abundance using NRMSE. We found that the NRMSE was significantly lower for some bacterial genera compared to others. This may suggest that certain genera experience large fluctuations in abundance, which could contribute to poorer prediction accuracy for these specific bacteria.

### Results for microbial communities from environmental samples

**Table 5:** Evaluation metrics for LSTM, GRU, and Random Forest models trained on the data obtained from the WWTP on Jones Island, Milwaukee^65^. Results for different variations of metadata inclusion. The LSTMs all consist of one layer and 2058 cells. The GRUs consist of one layer and 1024 cells, while the RF consists of 100 estimators. Overfitting can be observed for all models, as they are trained on only a subset of the data, and this can be tackled with training on the complete dataset.

Architecture	MAE training	MAE test	RMSE training	RMSE test	NRMSE training	NRMSE test
LSTM (no metadata)	15.06	74.20	92.99	459.39	0.12	0.95
LSTM (all metadata)	12.26	73.63	83.12	458.63	0.11	0.93
LSTM (prec+temp)	13.53	72.15	94.25	450.78	0.13	0.89
LSTM (chem. metadata)	16.47	70.81	108.10	454.72	0.16	1.07
RF (no metadata)	20.75	61.67	117.10	385.61	0.18	0.60
RF (all metadata)	21.07	62.18	119.36	386.92	0.18	0.65
RF (prec+temp)	20.97	62.95	119.31	395.83	0.18	0.63
Rf (chem. metadata)	20.67	63.58	118.80	392.11	0.18	0.65
GRU (all metadata)	19.06	74.18	99.49	464.27	0.13	1.11
GRU (no metadata)	15.40	71.08	80.18	450.89	0.11	1.00
GRU (prec+temp)	17.30	72.22	97.72	464.48	0.14	1.08
GRU (chem. metadata)	22.04	68.79	99.49	432.53	0.15	0.89

**Table 6:** Prediction results of the LSTM for time series data from the WWTP in Dinslaken, Germany. The addition of information about precipitation and air temperature does not improve the prediction accuracy.

Architecture	MAE training	MAE test	RMSE training	RMSE test	NRMSE training	NRMSE test
LSTM (no metadata)	24.39	142.51	202.99	887.71	0.14	1.35
LSTM (temp)	19.73	95.64	354.02	829.27	0.37	2.64
LSTM (prec+temp)	25.73	143.88	204.69	902.44	0.14	1.41

For the initial evaluation of the wastewater data, we used the same model architectures that were applied to the human data. Based on the performance differences observed with the intestinal microbiome data, we focused on the three best-performing models: LSTM, GRU, and Random Forest. These models were trained on microbial abundance data from LaMartina et al.^65^, specifically from the Jones Island dataset, using different combinations of additional metadata. For the LSTM model, comparing evaluation metrics such as MAE, RMSE, and NRMSE revealed that including additional metadata improved prediction accuracy (Table 5). This metadata included information on precipitation, temperature, flow (in million gallons per day), TSS (total suspended solids), and concentrations of ammonium, BOD5, and phosphorus^65^. The genus-specific predictions showed that while some bacterial genera were estimated accurately, predictions for others were less precise.

Environmental metadata (e.g., precipitation, temperature, chemical parameters) influenced performance variably. For instance, incorporating precipitation improved predictions for one WWTP but not for all, possibly due to infrastructural differences and how these factors influence community assembly and nutrient inputs.

In analyzing the SHAP output, we examined whether there was any correlation between bacterial abundances and the importance ranking of genera as determined by the model. Particular focus was given to the feature importance of known pathogens of interest, including the ESKAPE pathogens: *Enterococcus*, *Staphylococcus*, *Klebsiella*, *Acinetobacter*, *Pseudomonas*, and *Enterobacter*^96^. Although these genera were detected in samples from the Ruhr area, they did not rank among the most important features for the model’s predictions. Furthermore, no clear relationship was observed between the abundance of bacterial genera and their significance for the model’s performance. Similarly, a comparison of the features deemed most important to the model and those in the correlation network showed no apparent connection.

**Fig. 3:**
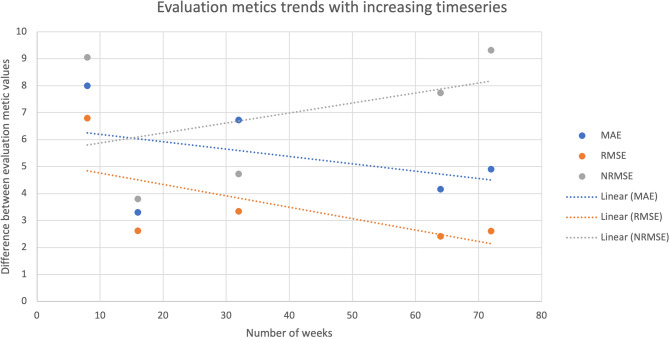
Difference between MAE, RMSE, and NRMSE for training and test set with different amounts of sampling points as input data. The scaling factor for MAE and RMSE between training and test sets decreases with a higher amount of input data. The increase in the difference between the NRMSE values could result from a shift in the data range. The overfitting of the model is reduced when training the model on a longer time series.

**Fig. 4:**
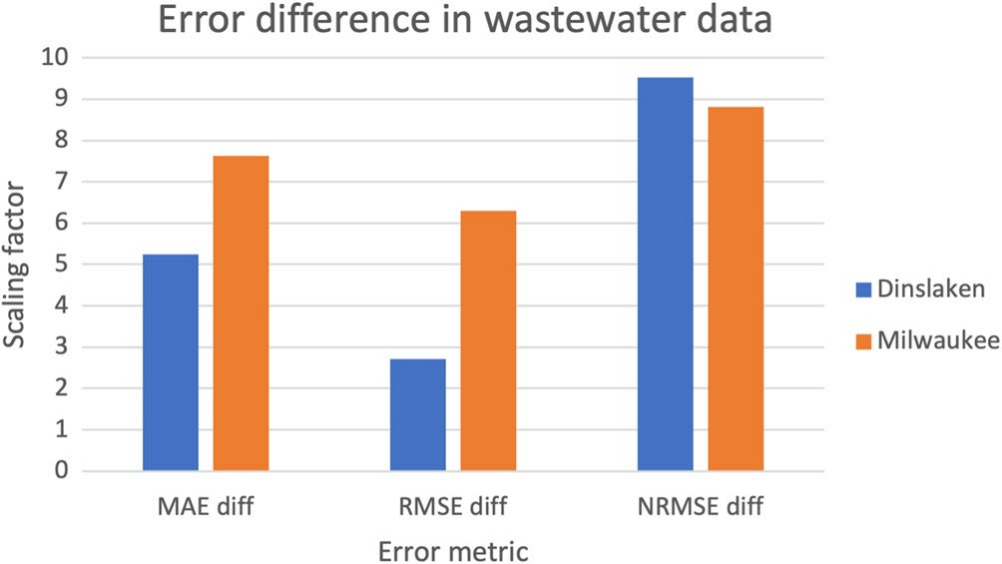
Differences in scaling factors between training and test evaluation metrics for the wastewater data from Milwaukee and Dinslaken. The deviation of evaluation metrics in training against test set for MAE and RMSE are smaller for the data from Dinslaken, the time series with a higher number of sampling points. This shows that a higher number of sampling points reduces the overfitting of a model.

To assess whether the length of the time series affects prediction accuracy, the weekly samples were divided into shorter time series of varying lengths. The model’s performance on these time series was evaluated by comparing changes in the evaluation metrics (Fig. 3). The results indicate that the differences between MAE and RMSE for the training and test sets decrease as the time series lengthens. In contrast, the NRMSE shows an increasing trend over time. This increase could reflect a shift in range or scale with additional data points and does not necessarily indicate reduced prediction accuracy. These changes in the evaluation metrics suggest that longer time series can help reduce overfitting. Additionally, training the model with more data can further reduce or eliminate overfitting, as shown in Appendix Fig. 10. Higher diversity in input data also seems to be beneficial to the models predictions, as can be seen when comparing the training results for human and wastewater data (Appendix Fig. 9). More frequent sampling intervals in the Dinslaken data reduced overfitting as well (Fig. 4), emphasizing the value of dense, longitudinal sampling for capturing community dynamics.

### Interpreting model predictions and community dynamics

**Fig. 5:**
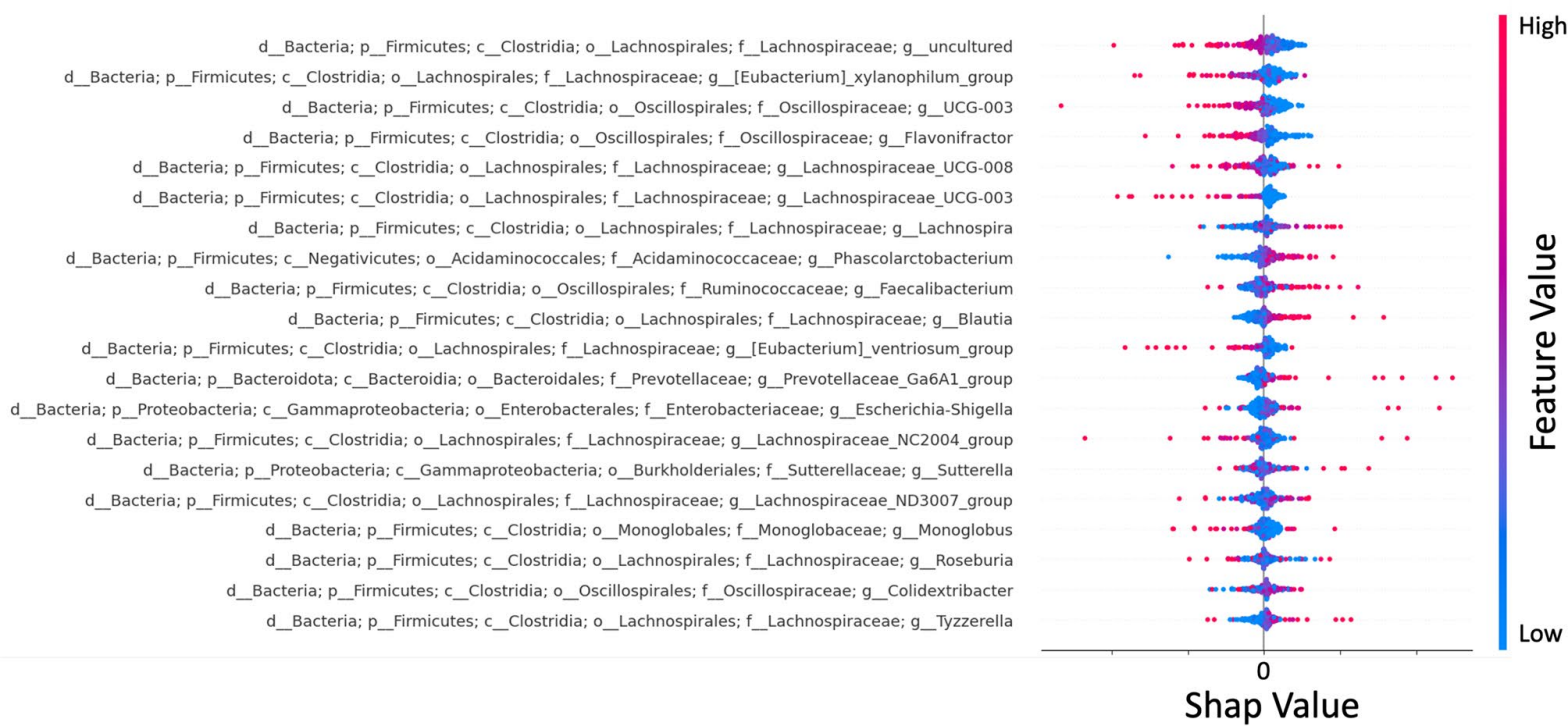
SHAP evaluation of the input parameter importance for intestinal microbiome of the female subject of Caporaso et al.^16^. The twenty most important bacterial genera are listed based on their feature importance, with the SHAP value computed for each time step. While the family of *Lachnospiraceae* have a high importance with a negative Shapley value, the genus *Lachnospira* is more important with a positive Shapley value.

To better understand the model’s predictions, we employed SHAP analysis^90^ to identify which genera contributed most strongly to abundance forecasts. We also explored correlations between taxa using SCNIC^91^.

**Fig. 6:**
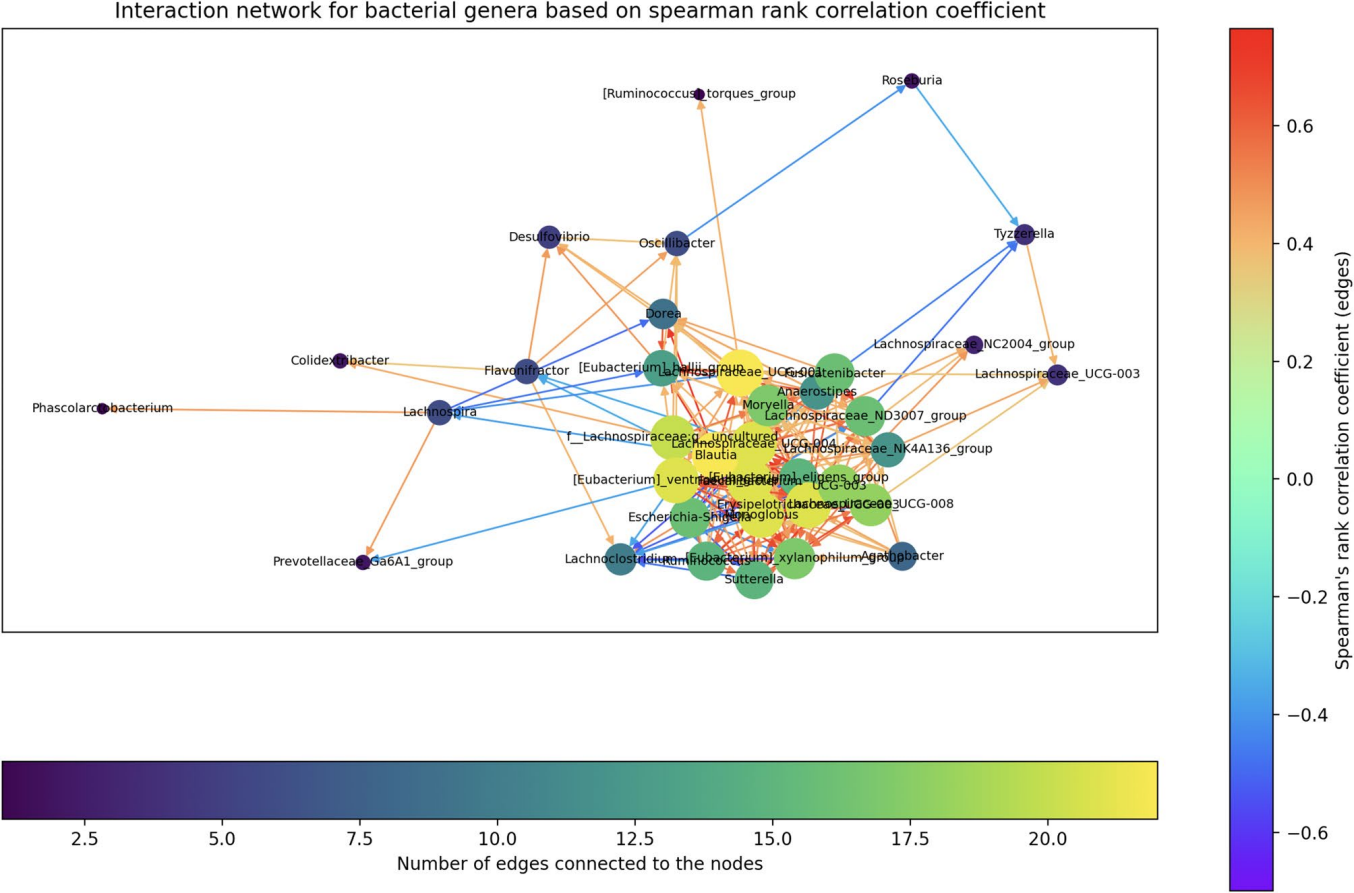
Depiction of network correlation analysis done with SCNIC for the intestinal microbiome of the female subject of Caporaso et al.^16^. The correlation was measured with Spearman’s rank correlation coefficient. The nodes’ color and size are set based on the number of connected nodes. Edges (depicted as arrows between nodes) are colored depending on the correlation coefficient. The genus with one of the most connections to other genera and the most connections with a Spearman’s rank correlation coefficient of greater than 0.6 is *Blautia*.

Key genera in this context are defined as those with a significant influence on the microbial community, as indicated by their strong correlations with other bacterial genera^97^. This approach is considered alongside other methods for identifying keystone genera^98,99^. To investigate this further, we compared the results from the SHAP analysis with those of the SCNIC analysis. With this, we can connect the importance of individual features for the model with the examined correlation between different genera. Figure 5 presents the most influential features for the final LSTM model based on the SHAP analysis. SCNIC calculations were performed for all individuals. These generated correlation networks illustrate both positive and negative relationships among bacterial genera (Fig. 6). Additional networks were constructed for modules of clustered genera, showing positive correlations between these clusters. Genera without any significant correlations were automatically excluded by SCNIC. When comparing SHAP-derived features with SCNIC results, we observed that the ten most influential features identified by SHAP were present in at least one of the top ten lists of significant bacteria in the correlation networks. Interesting results for both analyses were obtained for the genus *Blautia*. SHAP revealed *Blautia* as an important feature for the model’s predictive capability in case of a positive Shapley value. In addition, the network built with SCNIC based on Spearman’s rank correlation coefficient revealed an important role of *Blautia*. The abundance of *Blautia* has a strong positive correlation to other genera, making it the genus with the highest correlation coefficient in the network. This aligns with research suggesting that *Blautia* is connected with diseases like diabetes or obesity^100^ and plays a pivotal role in the gut microbiome^101^.

In the SCNIC analysis, the most significant bacteria were defined as those with the highest correlation coefficients with other genera or the largest number of connections within the network. While some highly influential taxa overlapped with strongly networked taxa, the model’s predictions were not solely driven by the most abundant or most connected genera. Instead, predictions emerged from the complex interplay of multiple genera and their temporal patterns.

## Discussion

To develop a model capable of predicting bacterial abundances over time and distinguishing between normal variations and outliers, we evaluated various machine learning methods and architectures using 16S rRNA gene amplicon sequencing datasets from human and environmental sources. The LSTM model with one hidden layer and 2048 cells was identified as the most effective, outperforming the GRU and Random Forest models for both dataset types.

The LSTM model demonstrated reliable performance in predicting bacterial abundances for both human and wastewater datasets, effectively identifying outliers using a prediction interval. However, the prediction accuracy was occasionally influenced by data-related limitations. Incorporating metadata into microbial time series data had varying effects on prediction outcomes, depending on the context. For instance, while including precipitation data improved model performance for the Milwaukee^65^ dataset, accuracy for the KLDI dataset from Germany declined with that kind of information (Table 5 and 6). These differences likely reflect the varying significance of rainfall in the respective wastewater systems. The KLDI treatment plant employs separate sewer systems for wastewater and rainwater, which explains why rainfall does not affect the machine learning model’s performance for this dataset. This finding highlights the importance of ensuring that added metadata is contextually relevant to enhance predictive performance.

The evaluation metrics for the different architectures reveal issues with underfitting and overfitting (Table 3). Our findings suggest that a model with a larger number of LSTM cells is required to capture patterns more accurately. However, the limited availability of data makes it challenging to train a large model without overfitting. Since our focus is on creating a prediction interval for outlier detection rather than precise point predictions, the impact of overfitting is less apparent in the abundance prediction plots. We also observed that overfitting can be mitigated by incorporating additional time points and retraining the model on larger datasets (Figs. 4 and 3, Appendix Fig. 10, Appendix Table 8). The problem of overfitting nevertheless requires careful model validation and hyperparameter tuning to ensure generalization.

The predictive power of the model is influenced not only by the amount of data available but also by the completeness of the datasets. Missing data points, such as those in the datasets from the Milwaukee and Chicago WWTPs, present a challenge. While the model can make predictions despite missing time points, imputing the gaps would provide a more comprehensive view of the microbiome’s dynamics. However, imputing missing data in microbial time series remains complex and requires further research^102^. Additionally, real-world applications might face challenges due to data scarcity, noise, or varying abundance levels across samples. Future research should focus on addressing these issues through improved data collection strategies, robust preprocessing methods, and advanced regularization techniques.

**Fig. 7:**
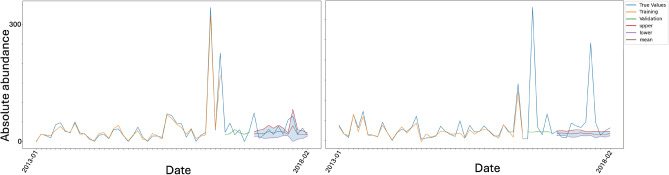
Predictions for different genera, left *Mycobacterium*, right *Bacteroidales* from the wastewater dataset from LaMartina *et al.*^65^. It becomes clear that the LSTM that predicts for one genus very well may as well predict abundances for another with less precision.

**Fig. 8:**
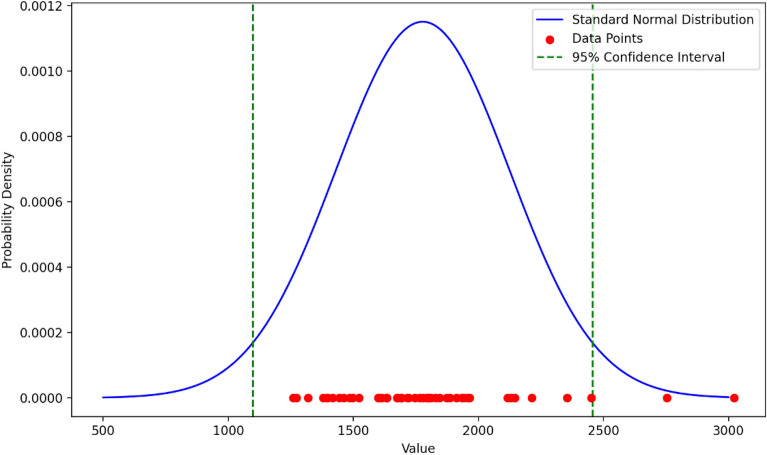
Graphical representation of how a standard normal distribution was used to calculate the 95% prediction interval for each bacterial genus at each time point. Based on the mean and the standard deviation of the predicted values, the standard normal distribution for them is calculated, followed by the calculation of the 95% confidence interval.

Challenges arose when retraining an already trained model on a dataset from a different sampling source. In these cases, some bacterial genera were predicted less accurately than others (Fig. 7). For the human datasets, the absence of health information for all participants made it difficult to determine whether these discrepancies were due to lifestyle factors (e.g., diet, travel) or potential medical events or if they represented true outliers. With data from only four individuals, the model is likely to learn some traits specific to the different sampled persons. Sadly, this kind of time series data is really scarce. However, the fact that we consider a prediction interval and do not make point predictions mitigates the impact of individual differences.

In the case of wastewater data, external factors not accounted for in the study, such as changes in chemical composition or extreme weather, could also explain the observed variations. Additional factors that may contribute to inaccuracies in LSTM predictions include limited training data, substantial differences between training and test datasets, or highly variable bacterial abundance peaks. The complexity of predicting microbial abundances is further compounded by the diversity of microbial environments and the varying abundance levels across samples.

While the challenges discussed above highlight areas for further research, the proposed method demonstrates the potential for achieving accurate predictions when long-term time series data is available. At least two time points are required for the model to generate predictions, as an initial understanding of community composition is necessary.

The findings of this study indicate that it is feasible to predict bacterial abundances over time in various environments using the presented machine learning model. This capability supports outlier detection, which can identify unusual changes in bacterial abundance. For this purpose, the model must handle short-term fluctuations and produce predictions with intervals rather than single-point estimates. To enhance predictive accuracy, models should first be trained on data from “normal” or stable conditions and subsequently updated with data from dysbiotic states.

Our predictive framework can serve as an early-stage analytical tool, prompting researchers to ask why an outlier emerged rather than just detecting that it did. By connecting predictions to host conditions, environmental parameters, or management interventions, this approach can inform targeted studies aimed at restoring microbial balance or mitigating adverse shifts. Ultimately, understanding when and why microbial communities deviate from their expected trajectories may improve our ability to maintain or restore healthy microbiomes across diverse ecosystems.

For example, in patient care, a dysbiosis in the microbiome that could potentially lead to a septic state may be identified, increasing the chances of early treatment. Such a prediction tool has potential applications across various fields. By applying our machine learning approach to monitor microbiome changes in ICU patients, clinicians could proactively address dysbiosis and reduce the risk of severe complications such as sepsis. Additionally, integrating our prediction model with electronic health records (EHRs) could facilitate personalized medicine by detecting dysbiosis and linking it to changes that would enable a timely intervention.

Our findings are not only limited to patient care but also have significant implications for environmental monitoring and public health. Wastewater epidemiology has emerged as a powerful tool for detecting and tracking infectious diseases^103^. By integrating our predictive modeling approach with wastewater analysis, we could improve early warning systems for identifying the potential growth of problematic bacteria^104^. This is critical for addressing public health challenges like waterborne disease outbreaks and climate change impacts on aquatic ecosystems. For wastewater, metadata plays a crucial role in prediction. The differential impact of temperature and precipitation metadata on prediction accuracy can be attributed to their distinct roles in shaping wastewater microbiomes. Temperature influences microbial growth rates and community composition, with optimal temperatures facilitating the proliferation of specific bacterial groups^64,65^. In contrast, precipitation primarily affects water volume and flow rates, which may indirectly influence microbial abundances through dilution or nutrient input^105^. By incorporating these environmental factors into our predictive models, we could better capture the complex dynamics driving wastewater microbiome fluctuations. In both scenarios, further analysis would be necessary to identify the cause of detected imbalances. Incorporating further relevant metadata, such as patient medical history, dietary factors, or environmental variables, is essential for accurately interpreting outliers and determining their underlying causes. This integration of predictive modeling and contextual information enhances the model’s utility for proactive decision-making.

## Conclusion and outlook

The findings of this study illustrate the potential of LSTM models to accurately predict microbial community composition in diverse environmental contexts. Despite the necessity for further research to address challenges such as missing data and limited information coverage, the model demonstrates the potential for applications in medical and public health contexts. The results of our study demonstrate that machine learning techniques, particularly LSTM models, can effectively predict microbial abundance trends in both human microbiomes and wastewater datasets. By establishing prediction intervals for each genus, we were able to detect outliers and identify critical shifts indicative of potential health risks or environmental stressors. These findings highlight the potential of predictive modeling in early detection and intervention for a wide range of applications, from personalized medicine to public health surveillance. To further advance our understanding of microbiome dynamics and improve predictive modeling, future research should prioritize the integration of diverse data types. Compared to 16S rRNA gene analysis, shotgun metagenomics offers a more detailed view of microbial communities, enabling the detection of lower-abundance species and functional profiling^106^. Additionally, exploring advanced machine learning techniques like deep learning or transfer learning could help enhance predictive performance across different datasets and environments.

## Figures and Tables

**Fig. 9:**
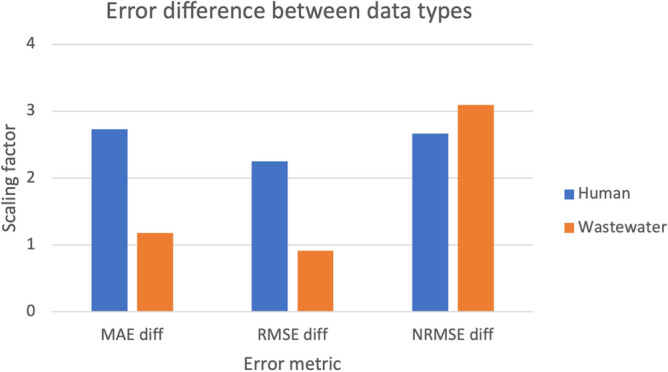
Difference between MAE, RMSE, and NRMSE for training and testing two data types: human and wastewater. The difference between the evaluation metrics is smaller for the wastewater samples than for the human samples, with the except for NRMSE. The increase in the scaling factor for the NRMSE could result from anomalies in the wastewater data or shifts in the values’ range. The graphic indicates that the training of the wastewater data was more successful than the training of the human data.

**Fig. 10:**
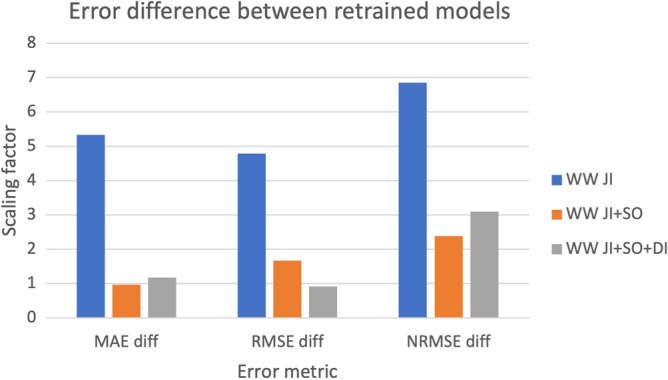
Difference between scaling factors for MAE, RMSE, and NRMSE for the model trained on different amounts of wastewater data. The model WW JI was trained only on the data from Jones Island, Milwaukee^65^. WW JI+SO was trained on data from Jones Island and then retrained on data from South Shore, Milwaukee^65^. The final model WW JI+SO+DI was trained on both datasets from Milwaukee and the data from Dinslaken. The overfitting for the first model is not visible anymore after retaining the model on additional data.

## Data Availability

The dataset from the KLDI used for training the machine learning model in this research has been deposited under the project accession PRJEB83882.

## Appendix

See Tables 7 and 8 and Figs. 7, 8, 9 and 10.

**Table 7:** Initial training of the different model architectures done with the other human datasets to verify that the LSTM is the best fit for the samples at hand when not training on the female dataset. It is clear, that for some evaluation metrics, the LSTM results not in the best values (especially for DonorA), but all together, the LSTM performs best in most cases.

	Male			DonorA			DonorB		
	LSTM	GRU	Random forest	LSTM	GRU	Random forest	LSTM	GRU	Random forest
MAE training	16.68	16.31	478.94	639.27	527.06	475.17	359.35	472.07	487.59
MAE test	66.52	158.76	1344.58	4984.53	5553.38	2245.52	1356.49	1811.36	2440.78
RMSE training	64.63	34.82	2360.21	2191.33	1710.21	2245.52	2262.85	1936.07	2440.78
RMSE test	208.66	375.62	4587.49	15870.72	15398.84	4383.76	4735.4	5867.49	4310.64
NRMSE training	0.25	0.12	0.19	0.13	0.1	0.18	0.19	0.14	0.2
NRMSE test	1.73	1.36	0.45	1.03	1.08	0.44	0.84	1.02	0.46

**Table 8:** Evaluation metric results for training the LSTM on the wastewater data from Jones Island and then retraining, first on the data from South Shore^65^ and then on the data from Dinslaken. While the problem of overfitting is tackled with additional data, the general prediction performance decreases.

Architecture	MAE training	MAE test	RMSE training	RMSE test	NRMSE training	NRMSE test
Jones Island	13.53	72.15	94.25	450.78	0.13	0.89
South Shore	39.25	37.84	308.79	514.29	1.01	2.40
Dinslaken	79.28	93.27	932.45	852.12	1.00	3.11
